# Bis(2,2′:6′,2′′-terpyridine)cobalt(II) bis­(tricyano­methanide)

**DOI:** 10.1107/S160053680901071X

**Published:** 2009-03-28

**Authors:** Jun Luo, Xin-Rong Zhang, Li-Juan Qiu, Bao-Shu Liu, Zhi-Yan Zhang

**Affiliations:** a School of Pharmacy, Second Military Medical University, Shanghai 200433, People’s Republic of China; b Department of Pharmacy, Changhai Hospital, Second Military Medical University, Shanghai 200433, People’s Republic of China

## Abstract

The title complex, [Co(C_15_H_11_N_3_)_2_](C_4_N_3_)_2_, is built up from discrete [Co(terpy)_2_]^2+^ cations (terpy is 2,2′:6′,2′′-terpyridine) and [C(CN)_3_]^−^ anions. In the cation, the Co^II^ atom is coordinated by two terpy mol­ecules, giving a distorted octa­hedral geometry. The tricyano­methanide anions are not directly coordinated to the Co^II^ atom, but some weak C—H⋯N hydrogen bonds involving the terminal N atoms of the tricyaomethanide ions and the terpyridine H atoms link anions and cations building a three-dimensional network.

## Related literature

For the structural characteristics and magnetic properties of tricyano­methanide coordination polymers, see: Batten *et al.* (1998 ▶, 2000 ▶); Batten & Murray (2003 ▶); Miller & Manson (2001 ▶); Manson *et al.* (1998 ▶, 2000 ▶); Manson & Schlueter (2004 ▶); Feyerherm *et al.* (2003 ▶, 2004 ▶); Abrahams *et al.* (2003 ▶); Hoshino *et al.* (1999 ▶); Yuste *et al.* (2008 ▶); Luo *et al.* (2008 ▶). For Co—N(terpy) distances in other cobalt–terpyridine complexes, see: Indumathy *et al.* (2007 ▶). For bond distances and bond angles in other tricyano­methanide complexes, see: Hoshino *et al.* (1999 ▶); Batten *et al.* (1999 ▶). For weak C—H⋯N inter­actions, see: Nardelli (1995 ▶).
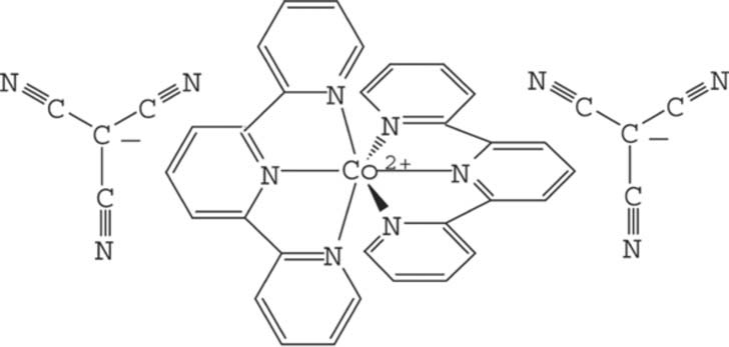


## Experimental

### 

#### Crystal data

[Co(C_15_H_11_N_3_)_2_](C_4_N_3_)_2_
*M* *_r_* = 705.61
Monoclinic, 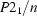

*a* = 9.042 (3) Å
*b* = 9.167 (3) Å
*c* = 40.340 (14) Å
β = 91.163 (6)°
*V* = 3343 (2) Å^3^
*Z* = 4
Mo *K*α radiation
μ = 0.56 mm^−1^
*T* = 293 K
0.20 × 0.15 × 0.10 mm

#### Data collection

Bruker SMART APEX CCD area-detector diffractometer
Absorption correction: multi-scan (*SADABS*; Sheldrick, 1996 ▶) *T*_min_ = 0.896, *T*_max_ = 0.946
13582 measured reflections
5880 independent reflections
3009 reflections with *I* > 2σ(*I*)
*R*_int_ = 0.093

#### Refinement

*R*[*F* ^2^ > 2σ(*F* ^2^)] = 0.061
*wR*(*F* ^2^) = 0.106
*S* = 0.96
5880 reflections
460 parameters
H-atom parameters constrained
Δρ_max_ = 0.27 e Å^−3^
Δρ_min_ = −0.20 e Å^−3^

### 

Data collection: *SMART* (Bruker, 2000 ▶); cell refinement: *SAINT* (Bruker, 2000 ▶); data reduction: *SAINT*; program(s) used to solve structure: *SHELXS97* (Sheldrick, 2008 ▶); program(s) used to refine structure: *SHELXL97* (Sheldrick, 2008 ▶); molecular graphics: *PLATON* (Spek, 2009 ▶); software used to prepare material for publication: *SHELXL97*

## Supplementary Material

Crystal structure: contains datablocks global, I. DOI: 10.1107/S160053680901071X/dn2438sup1.cif

Structure factors: contains datablocks I. DOI: 10.1107/S160053680901071X/dn2438Isup2.hkl

Additional supplementary materials:  crystallographic information; 3D view; checkCIF report

## Figures and Tables

**Table 1:** Hydrogen-bond geometry (Å, °)

*D*—H⋯*A*	*D*—H	H⋯*A*	*D*⋯*A*	*D*—H⋯*A*
C4—H4⋯N7	0.93	2.74	3.589 (7)	153
C8—H8⋯N9	0.93	2.71	3.347 (8)	126
C15—H15⋯N11	0.93	2.55	3.254 (6)	133
C1—H1⋯N12^i^	0.93	2.85	3.598 (6)	138
C23—H23⋯N11^i^	0.93	2.89	3.622 (6)	136
C2—H2⋯N10^ii^	0.93	2.77	3.670 (7)	164
C29—H29⋯N9^iii^	0.93	2.85	3.560 (8)	134
C17—H17⋯N8^iv^	0.93	2.65	3.395 (7)	137
C13—H13⋯N8^v^	0.93	2.65	3.379 (7)	136
C18—H18⋯N12^vi^	0.93	2.69	3.403 (6)	134
C22—H22⋯N10^vi^	0.93	2.51	3.231 (6)	134
C19—H19⋯N12^vii^	0.93	2.96	3.679 (6)	135
C22—H22⋯N12^vii^	0.93	2.92	3.645 (6)	136
C24—H24⋯N10^viii^	0.93	2.67	3.548 (6)	158
C27—H27⋯N10^viii^	0.93	2.57	3.350 (6)	142

Symmetry codes: (i) 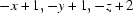; (ii) 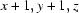; (iii) 
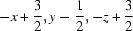
; (iv) 
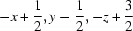
; (v) 
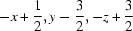
; (vi) 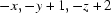; (vii) 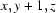; (viii) 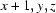.

**Fig. 1.**
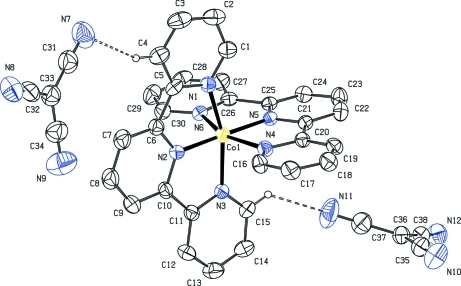
A view of the cation-anion pair in (I), showing the atom-labelling scheme. Displacement ellipsoids are drawn at the 30% probability level. H atoms not involved in hydrogen bondings have been omitted for clarity. H bonds are shown as dashed lines.

## supplementary crystallographic information

### Comment

Recently, coordination polymers constructed by tricyanomethanide (tcm) have attracted considerable interest due to their unique structure characteristics and fascinating magnetic properties (Batten *et al.*, 2003; Miller *et al.*, 2001; Feyerherm *et al.*, 2003). Interestingly, most binary tcm complexes reveal a rutile-like structure (Manson *et al.*, 2000, 1998; Hoshino *et al.*, 1999; Feyerherm *et al.*, 2004), except that a doubly interpenetrated (6,3) sheet was detected in Ag(tcm)_2_ (Abrahams *et al.*, 2003). To elucidate the structure-properties relationship of tcm complexes, diverse co-ligands such as hexamethylenetetramine, 4,4-bipyridyl, 1,2-bi(4-pyridyl)ethane were introduced and the structures as well as magnetic properties of the modified complexes have been systematically investigated. Among the Cu(I) or Cd(II) tcm complexes with these co-ligands, numerous structure types range from doubly interpenetrated (4,4) sheet to three-dimensional rutile networks were observed (Batten *et al.*, 2000, 1998). By contrast, adjustment of the Mn(II)-tcm binary system with 4,4-bipyridyl as co-ligands leads to the formation of a one dimensional chain-like structure (Manson *et al.*, 2004). On the other hand, 2,2':6'2''-terpyridine (terpy) has three potential nitrogen donor atoms. However, a few tcm complexes with terpy as a co-ligand have ever been reported (Yuste *et al.*, 2008; Luo *et al.*, 2008). To further study the role of the nature of co-ligands on the structures and properties of tricyanomethanide complexes, we herein report the synthesis and crystal structure of the new tricyanomethanide complex [Co(terpy)_2_](C_4_N_3_) _2_ (I).

In I the cobalt ion is bonded to six N atoms from two terpyridine molecules to define the cation part, in which the basal plane is formed by the three N atoms (N1, N2, N3) of one terpy ligand and one N atom (N5) of the other terpy ligand, the apical sites are occupied by two N atoms (N4 and N6) of the latter terpy ligand. The tricyanomethanide anions do not enter the inner coordination sphere, but are linked to the cation part *via* weak C-H···N interactions (Fig. 1). These weak C-H···N interactions (Nardelli, 1995) build up a three dimensional network (Table 1).

In I, the Co—N(terpy) distances are in the range from 1.858 (3)Å to 2.139 (3) Å, these value are similar to the corresponding distances observed in other cobalt-terpyridine complexes (Indumathy *et al.*, 2007).

Each tricyanomethanide moiety is almost planar. Bond distances and bond angles within the anions are in good agreement with those found in other tricyanomethanide complexes (Hoshino *et al.*, 1999; Batten *et al.*, 1999).

### Experimental

A 5 ml e thanol solution of terpyridine (0.10 mmol, 23.33 mg) and a 2 ml aqueous pink solution of cobalt nitrate (0.10 mmol, 29.10 mg) were mixed and stirred for 5 min, the mixed solution was deep-brown. To the mixture was added a 3 ml e thanol-water solution (EtOH:H_2_O = 2:1, V:V) of potassium tricyanomethanide (0.20 mmol, 25.83 mg). After stirring for another 5 min, the deep-brown solution was filtered and the filtrate was slowly evaporated in air. After two week, deep-brown block crystals of I were isolated in 17% yield. Anal: Calculated for C38H22CoN12: C 64.68%, H 3.14%, N 23.82%. Found C 64.84%, H 3.22%, N 23.95%.

### Refinement

The H atoms were treated as riding on their parent atoms with C—H distances of 0.93 Å and U_iso_(H) = 1.2U_eq_(C).

### Crystal data

**Table:**

[Co(C_15_H_11_N_3_)_2_](C_4_N_3_)_2_	*F*(000) = 1444
*M**_r_* = 705.61	*D*_x_ = 1.402 Mg m^−^^3^
Monoclinic, *P*2_1_/*n*	Mo *K*α radiation, λ = 0.71073 Å
Hall symbol: -P 2yn	Cell parameters from 925 reflections
*a* = 9.042 (3) Å	θ = 2.3–17.9°
*b* = 9.167 (3) Å	µ = 0.56 mm^−^^1^
*c* = 40.340 (14) Å	*T* = 293 K
β = 91.163 (6)°	Block, dark-brown
*V* = 3343 (2) Å^3^	0.20 × 0.15 × 0.10 mm
*Z* = 4	

### Data collection

**Table:**

Bruker SMART APEX CCD area-detector diffractometer	5880 independent reflections
Radiation source: fine-focus sealed tube	3009 reflections with *I* > 2σ(*I*)
graphite	*R*_int_ = 0.093
φ and ω scans	θ_max_ = 25.0°, θ_min_ = 1.0°
Absorption correction: multi-scan (*SADABS*; Sheldrick, 1996)	*h* = −10→10
*T*_min_ = 0.896, *T*_max_ = 0.946	*k* = −10→7
13582 measured reflections	*l* = −41→47

### Refinement

**Table:**

Refinement on *F*^2^	Primary atom site location: structure-invariant direct methods
Least-squares matrix: full	Secondary atom site location: difference Fourier map
*R*[*F*^2^ > 2σ(*F*^2^)] = 0.061	Hydrogen site location: inferred from neighbouring sites
*wR*(*F*^2^) = 0.106	H-atom parameters constrained
*S* = 0.96	*w* = 1/[σ^2^(*F*_o_^2^) + (0.0281*P*)^2^] where *P* = (*F*_o_^2^ + 2*F*_c_^2^)/3
5880 reflections	(Δ/σ)_max_ = 0.001
460 parameters	Δρ_max_ = 0.27 e Å^−^^3^
0 restraints	Δρ_min_ = −0.20 e Å^−^^3^

### Special details

**Table:**

Geometry. All esds (except the esd in the dihedral angle between two l.s. planes) are estimated using the full covariance matrix. The cell esds are taken into account individually in the estimation of esds in distances, angles and torsion angles; correlations between esds in cell parameters are only used when they are defined by crystal symmetry. An approximate (isotropic) treatment of cell esds is used for estimating esds involving l.s. planes.
Refinement. Refinement of *F*^2^ against ALL reflections. The weighted *R*-factor *wR* and goodness of fit *S* are based on *F*^2^, conventional *R*-factors *R* are based on *F*, with *F* set to zero for negative *F*^2^. The threshold expression of *F*^2^ > σ(*F*^2^) is used only for calculating *R*-factors(gt) *etc*. and is not relevant to the choice of reflections for refinement. *R*-factors based on *F*^2^ are statistically about twice as large as those based on *F*, and *R*- factors based on ALL data will be even larger.

### Fractional atomic coordinates and isotropic or equivalent isotropic displacement parameters (Å^2^)

**Table:**

	*x*	*y*	*z*	*U*_iso_*/*U*_eq_	
Co1	0.50103 (6)	0.82076 (6)	0.873406 (12)	0.04411 (18)	
N1	0.5876 (3)	1.0222 (4)	0.87728 (8)	0.0463 (9)	
N2	0.4973 (3)	0.8719 (4)	0.82884 (7)	0.0448 (9)	
N3	0.4127 (3)	0.6366 (3)	0.85446 (8)	0.0442 (9)	
N4	0.2901 (4)	0.9020 (3)	0.88713 (8)	0.0481 (9)	
N5	0.4994 (4)	0.7677 (3)	0.91878 (7)	0.0390 (8)	
N6	0.7138 (3)	0.7206 (3)	0.87885 (8)	0.0463 (9)	
N7	0.7089 (6)	1.5090 (7)	0.78492 (11)	0.125 (2)	
N8	0.5165 (6)	1.6925 (6)	0.69117 (11)	0.1123 (18)	
N9	0.4373 (7)	1.2455 (7)	0.71648 (15)	0.143 (2)	
N10	−0.1268 (5)	0.3628 (5)	0.98032 (11)	0.0990 (16)	
N11	0.3099 (6)	0.3292 (7)	0.93731 (12)	0.134 (2)	
N12	0.1971 (5)	0.0363 (5)	1.01956 (9)	0.0799 (13)	
C1	0.6312 (5)	1.0933 (5)	0.90478 (11)	0.0591 (12)	
H1	0.6209	1.0478	0.9252	0.071*	
C2	0.6907 (5)	1.2314 (6)	0.90391 (13)	0.0734 (15)	
H2	0.7200	1.2783	0.9234	0.088*	
C3	0.7061 (5)	1.2981 (5)	0.87393 (15)	0.0823 (16)	
H3	0.7464	1.3913	0.8728	0.099*	
C4	0.6618 (5)	1.2270 (5)	0.84551 (12)	0.0665 (14)	
H4	0.6721	1.2713	0.8249	0.080*	
C5	0.6023 (4)	1.0899 (5)	0.84780 (11)	0.0495 (11)	
C6	0.5480 (4)	1.0029 (5)	0.81958 (10)	0.0502 (11)	
C7	0.5449 (5)	1.0430 (5)	0.78661 (11)	0.0687 (14)	
H7	0.5823	1.1327	0.7801	0.082*	
C8	0.4850 (5)	0.9473 (6)	0.76338 (11)	0.0746 (15)	
H8	0.4813	0.9730	0.7411	0.090*	
C9	0.4313 (5)	0.8148 (6)	0.77329 (10)	0.0677 (13)	
H9	0.3899	0.7505	0.7579	0.081*	
C10	0.4397 (4)	0.7784 (5)	0.80649 (10)	0.0506 (12)	
C11	0.3901 (4)	0.6421 (5)	0.82150 (10)	0.0483 (11)	
C12	0.3275 (5)	0.5262 (5)	0.80430 (11)	0.0651 (13)	
H12	0.3131	0.5316	0.7814	0.078*	
C13	0.2866 (5)	0.4027 (5)	0.82127 (13)	0.0782 (15)	
H13	0.2444	0.3240	0.8100	0.094*	
C14	0.3089 (5)	0.3974 (5)	0.85490 (13)	0.0686 (14)	
H14	0.2815	0.3157	0.8670	0.082*	
C15	0.3725 (4)	0.5155 (5)	0.87038 (11)	0.0553 (12)	
H15	0.3887	0.5111	0.8932	0.066*	
C16	0.1884 (5)	0.9738 (5)	0.86914 (10)	0.0570 (12)	
H16	0.2067	0.9920	0.8469	0.068*	
C17	0.0580 (5)	1.0218 (5)	0.88197 (11)	0.0650 (13)	
H17	−0.0096	1.0734	0.8688	0.078*	
C18	0.0289 (5)	0.9927 (5)	0.91433 (12)	0.0665 (14)	
H18	−0.0597	1.0226	0.9235	0.080*	
C19	0.1318 (5)	0.9189 (5)	0.93328 (10)	0.0569 (12)	
H19	0.1140	0.8988	0.9554	0.068*	
C20	0.2617 (5)	0.8748 (4)	0.91917 (10)	0.0447 (11)	
C21	0.3810 (5)	0.7973 (4)	0.93730 (10)	0.0441 (10)	
C22	0.3758 (5)	0.7565 (5)	0.97017 (10)	0.0575 (12)	
H22	0.2949	0.7805	0.9829	0.069*	
C23	0.4929 (6)	0.6797 (5)	0.98366 (10)	0.0651 (13)	
H23	0.4913	0.6513	1.0058	0.078*	
C24	0.6118 (5)	0.6446 (4)	0.96480 (10)	0.0521 (12)	
H24	0.6889	0.5884	0.9735	0.063*	
C25	0.6151 (4)	0.6943 (4)	0.93254 (10)	0.0436 (10)	
C26	0.7392 (4)	0.6738 (4)	0.91018 (10)	0.0434 (10)	
C27	0.8725 (5)	0.6127 (4)	0.91970 (11)	0.0582 (12)	
H27	0.8894	0.5840	0.9416	0.070*	
C28	0.9802 (5)	0.5945 (5)	0.89658 (13)	0.0671 (14)	
H28	1.0700	0.5516	0.9026	0.081*	
C29	0.9545 (5)	0.6399 (5)	0.86442 (12)	0.0709 (14)	
H29	1.0257	0.6282	0.8483	0.085*	
C30	0.8198 (5)	0.7032 (5)	0.85694 (10)	0.0578 (12)	
H30	0.8022	0.7355	0.8354	0.069*	
C31	0.6441 (6)	1.4961 (6)	0.76014 (16)	0.0919 (18)	
C32	0.5614 (6)	1.4782 (7)	0.73069 (14)	0.0798 (16)	
C33	0.5377 (6)	1.5952 (8)	0.70963 (15)	0.0877 (18)	
C34	0.4954 (7)	1.3530 (9)	0.72316 (15)	0.095 (2)	
C35	−0.0143 (7)	0.3069 (6)	0.98015 (11)	0.0685 (14)	
C36	0.1248 (6)	0.2411 (5)	0.97914 (11)	0.0605 (13)	
C37	0.2264 (7)	0.2885 (6)	0.95613 (13)	0.0851 (17)	
C38	0.1641 (5)	0.1271 (6)	1.00156 (12)	0.0623 (14)	

### Atomic displacement parameters (Å^2^)

**Table:**

	*U*^11^	*U*^22^	*U*^33^	*U*^12^	*U*^13^	*U*^23^
Co1	0.0432 (4)	0.0457 (3)	0.0434 (3)	0.0000 (3)	0.0007 (2)	0.0009 (3)
N1	0.043 (2)	0.051 (2)	0.044 (2)	0.0019 (18)	−0.0023 (17)	0.0019 (19)
N2	0.043 (2)	0.049 (2)	0.043 (2)	−0.0044 (18)	−0.0004 (17)	−0.0033 (18)
N3	0.040 (2)	0.049 (2)	0.044 (2)	−0.0012 (17)	0.0030 (17)	−0.0019 (18)
N4	0.047 (2)	0.052 (2)	0.045 (2)	0.0024 (18)	0.0013 (18)	−0.0024 (18)
N5	0.038 (2)	0.038 (2)	0.041 (2)	−0.0004 (17)	0.0005 (17)	0.0006 (16)
N6	0.043 (2)	0.047 (2)	0.049 (2)	−0.0055 (17)	0.0023 (18)	0.0000 (18)
N7	0.109 (5)	0.181 (6)	0.084 (4)	0.013 (4)	−0.007 (3)	0.006 (4)
N8	0.137 (5)	0.114 (5)	0.087 (4)	0.011 (4)	0.004 (3)	−0.002 (3)
N9	0.137 (6)	0.116 (5)	0.176 (6)	−0.024 (4)	−0.028 (4)	0.016 (4)
N10	0.082 (4)	0.086 (4)	0.129 (4)	0.016 (3)	0.010 (3)	0.036 (3)
N11	0.106 (4)	0.194 (6)	0.104 (4)	−0.018 (4)	0.023 (3)	0.068 (4)
N12	0.090 (4)	0.076 (3)	0.073 (3)	0.004 (3)	0.001 (2)	0.013 (2)
C1	0.054 (3)	0.063 (3)	0.060 (3)	0.004 (3)	−0.008 (2)	0.003 (3)
C2	0.062 (4)	0.062 (4)	0.097 (4)	−0.002 (3)	−0.010 (3)	−0.019 (3)
C3	0.073 (4)	0.050 (3)	0.123 (5)	−0.010 (3)	−0.008 (3)	−0.008 (4)
C4	0.062 (4)	0.053 (3)	0.084 (4)	−0.003 (3)	−0.003 (3)	0.009 (3)
C5	0.036 (3)	0.043 (3)	0.069 (3)	−0.001 (2)	0.000 (2)	0.011 (3)
C6	0.048 (3)	0.056 (3)	0.046 (3)	0.002 (2)	0.000 (2)	0.003 (3)
C7	0.076 (4)	0.071 (4)	0.059 (3)	−0.006 (3)	0.004 (3)	0.019 (3)
C8	0.076 (4)	0.096 (4)	0.053 (3)	−0.001 (3)	0.002 (3)	0.015 (3)
C9	0.075 (4)	0.082 (4)	0.046 (3)	−0.008 (3)	−0.003 (2)	0.002 (3)
C10	0.052 (3)	0.063 (3)	0.037 (3)	0.001 (2)	0.001 (2)	0.002 (2)
C11	0.042 (3)	0.055 (3)	0.047 (3)	0.004 (2)	0.002 (2)	−0.007 (2)
C12	0.069 (4)	0.071 (4)	0.055 (3)	−0.009 (3)	−0.002 (3)	−0.018 (3)
C13	0.078 (4)	0.068 (4)	0.089 (4)	−0.020 (3)	−0.003 (3)	−0.014 (3)
C14	0.070 (4)	0.053 (3)	0.082 (4)	−0.015 (3)	0.001 (3)	0.002 (3)
C15	0.048 (3)	0.058 (3)	0.060 (3)	−0.005 (3)	0.007 (2)	0.004 (3)
C16	0.059 (4)	0.065 (3)	0.047 (3)	0.010 (3)	0.002 (3)	0.003 (2)
C17	0.055 (4)	0.077 (4)	0.063 (3)	0.015 (3)	−0.011 (3)	0.002 (3)
C18	0.048 (3)	0.085 (4)	0.066 (3)	0.013 (3)	0.003 (3)	−0.012 (3)
C19	0.050 (3)	0.071 (3)	0.050 (3)	0.005 (3)	0.000 (3)	−0.015 (2)
C20	0.043 (3)	0.045 (3)	0.047 (3)	0.003 (2)	0.002 (2)	−0.005 (2)
C21	0.049 (3)	0.040 (3)	0.043 (3)	−0.004 (2)	0.003 (2)	0.000 (2)
C22	0.061 (3)	0.059 (3)	0.053 (3)	0.001 (3)	0.013 (2)	0.004 (2)
C23	0.072 (4)	0.074 (3)	0.050 (3)	0.003 (3)	0.004 (3)	0.014 (3)
C24	0.059 (3)	0.044 (3)	0.053 (3)	0.002 (2)	−0.005 (2)	0.007 (2)
C25	0.044 (3)	0.038 (2)	0.048 (3)	−0.006 (2)	0.000 (2)	−0.002 (2)
C26	0.041 (3)	0.036 (2)	0.053 (3)	0.001 (2)	−0.006 (2)	−0.002 (2)
C27	0.048 (3)	0.060 (3)	0.066 (3)	0.002 (3)	−0.003 (3)	0.003 (2)
C28	0.044 (3)	0.059 (3)	0.098 (4)	0.006 (2)	−0.001 (3)	0.000 (3)
C29	0.056 (4)	0.071 (4)	0.085 (4)	0.003 (3)	0.017 (3)	0.001 (3)
C30	0.061 (3)	0.055 (3)	0.058 (3)	−0.005 (3)	0.009 (3)	−0.001 (2)
C31	0.078 (5)	0.112 (5)	0.086 (5)	0.005 (4)	0.013 (4)	−0.001 (4)
C32	0.072 (4)	0.095 (5)	0.072 (4)	0.001 (4)	0.000 (3)	0.008 (4)
C33	0.088 (5)	0.107 (6)	0.068 (4)	0.001 (4)	0.002 (4)	−0.021 (4)
C34	0.075 (5)	0.114 (7)	0.096 (5)	−0.006 (4)	−0.007 (4)	0.008 (5)
C35	0.082 (4)	0.056 (4)	0.068 (3)	−0.003 (3)	0.002 (3)	0.014 (3)
C36	0.069 (4)	0.060 (3)	0.053 (3)	−0.006 (3)	−0.001 (3)	0.011 (3)
C37	0.081 (4)	0.102 (5)	0.072 (4)	−0.003 (4)	−0.011 (3)	0.024 (3)
C38	0.065 (4)	0.067 (4)	0.056 (3)	−0.009 (3)	0.005 (3)	−0.007 (3)

### Geometric parameters (Å, °)

**Table:**

Co1—N2	1.858 (3)	C10—C11	1.463 (5)
Co1—N5	1.894 (3)	C11—C12	1.384 (5)
Co1—N1	2.011 (3)	C12—C13	1.377 (6)
Co1—N3	2.012 (3)	C12—H12	0.9300
Co1—N4	2.131 (3)	C13—C14	1.368 (5)
Co1—N6	2.139 (3)	C13—H13	0.9300
N1—C1	1.339 (5)	C14—C15	1.370 (5)
N1—C5	1.350 (5)	C14—H14	0.9300
N2—C6	1.341 (5)	C15—H15	0.9300
N2—C10	1.342 (4)	C16—C17	1.370 (5)
N3—C15	1.337 (5)	C16—H16	0.9300
N3—C11	1.342 (4)	C17—C18	1.363 (5)
N4—C16	1.334 (4)	C17—H17	0.9300
N4—C20	1.346 (4)	C18—C19	1.371 (5)
N5—C21	1.346 (4)	C18—H18	0.9300
N5—C25	1.354 (4)	C19—C20	1.376 (5)
N6—C30	1.326 (5)	C19—H19	0.9300
N6—C26	1.350 (4)	C20—C21	1.473 (5)
N7—C31	1.155 (6)	C21—C22	1.379 (5)
N8—C33	1.176 (7)	C22—C23	1.375 (5)
N9—C34	1.146 (7)	C22—H22	0.9300
N10—C35	1.139 (6)	C23—C24	1.368 (5)
N11—C37	1.144 (6)	C23—H23	0.9300
N12—C38	1.141 (5)	C24—C25	1.379 (5)
C1—C2	1.376 (6)	C24—H24	0.9300
C1—H1	0.9300	C25—C26	1.466 (5)
C2—C3	1.365 (6)	C26—C27	1.377 (5)
C2—H2	0.9300	C27—C28	1.372 (5)
C3—C4	1.371 (5)	C27—H27	0.9300
C3—H3	0.9300	C28—C29	1.378 (5)
C4—C5	1.371 (5)	C28—H28	0.9300
C4—H4	0.9300	C29—C30	1.377 (6)
C5—C6	1.466 (5)	C29—H29	0.9300
C6—C7	1.380 (5)	C30—H30	0.9300
C7—C8	1.385 (6)	C31—C32	1.400 (7)
C7—H7	0.9300	C32—C34	1.327 (8)
C8—C9	1.370 (6)	C32—C33	1.382 (7)
C8—H8	0.9300	C35—C36	1.396 (6)
C9—C10	1.381 (5)	C36—C37	1.389 (7)
C9—H9	0.9300	C36—C38	1.423 (6)
			
N2—Co1—N5	178.48 (14)	C11—C12—H12	120.2
N2—Co1—N1	80.96 (14)	C14—C13—C12	119.1 (4)
N5—Co1—N1	99.85 (13)	C14—C13—H13	120.5
N2—Co1—N3	81.07 (14)	C12—C13—H13	120.5
N5—Co1—N3	98.11 (13)	C13—C14—C15	118.4 (4)
N1—Co1—N3	162.03 (14)	C13—C14—H14	120.8
N2—Co1—N4	99.46 (14)	C15—C14—H14	120.8
N5—Co1—N4	79.28 (14)	N3—C15—C14	123.6 (4)
N1—Co1—N4	90.45 (12)	N3—C15—H15	118.2
N3—Co1—N4	92.38 (12)	C14—C15—H15	118.2
N2—Co1—N6	101.91 (13)	N4—C16—C17	122.8 (4)
N5—Co1—N6	79.36 (13)	N4—C16—H16	118.6
N1—Co1—N6	92.19 (12)	C17—C16—H16	118.6
N3—Co1—N6	91.61 (12)	C18—C17—C16	118.9 (4)
N4—Co1—N6	158.61 (13)	C18—C17—H17	120.6
C1—N1—C5	118.3 (4)	C16—C17—H17	120.6
C1—N1—Co1	128.3 (3)	C17—C18—C19	119.3 (4)
C5—N1—Co1	113.5 (3)	C17—C18—H18	120.4
C6—N2—C10	121.0 (3)	C19—C18—H18	120.4
C6—N2—Co1	119.7 (3)	C18—C19—C20	119.3 (4)
C10—N2—Co1	119.2 (3)	C18—C19—H19	120.4
C15—N3—C11	118.0 (3)	C20—C19—H19	120.4
C15—N3—Co1	128.6 (3)	N4—C20—C19	121.6 (4)
C11—N3—Co1	113.4 (3)	N4—C20—C21	114.4 (4)
C16—N4—C20	118.1 (4)	C19—C20—C21	123.9 (4)
C16—N4—Co1	129.9 (3)	N5—C21—C22	121.5 (4)
C20—N4—Co1	112.0 (3)	N5—C21—C20	113.8 (4)
C21—N5—C25	119.3 (3)	C22—C21—C20	124.7 (4)
C21—N5—Co1	120.5 (3)	C23—C22—C21	118.6 (4)
C25—N5—Co1	120.2 (3)	C23—C22—H22	120.7
C30—N6—C26	118.3 (4)	C21—C22—H22	120.7
C30—N6—Co1	130.1 (3)	C24—C23—C22	120.5 (4)
C26—N6—Co1	111.6 (3)	C24—C23—H23	119.7
N1—C1—C2	122.4 (4)	C22—C23—H23	119.7
N1—C1—H1	118.8	C23—C24—C25	118.6 (4)
C2—C1—H1	118.8	C23—C24—H24	120.7
C3—C2—C1	118.8 (5)	C25—C24—H24	120.7
C3—C2—H2	120.6	N5—C25—C24	121.3 (4)
C1—C2—H2	120.6	N5—C25—C26	114.0 (4)
C2—C3—C4	119.6 (5)	C24—C25—C26	124.7 (4)
C2—C3—H3	120.2	N6—C26—C27	121.5 (4)
C4—C3—H3	120.2	N6—C26—C25	114.7 (4)
C5—C4—C3	119.2 (5)	C27—C26—C25	123.8 (4)
C5—C4—H4	120.4	C28—C27—C26	119.3 (4)
C3—C4—H4	120.4	C28—C27—H27	120.4
N1—C5—C4	121.7 (4)	C26—C27—H27	120.4
N1—C5—C6	113.4 (4)	C27—C28—C29	119.7 (4)
C4—C5—C6	124.9 (4)	C27—C28—H28	120.1
N2—C6—C7	120.4 (4)	C29—C28—H28	120.1
N2—C6—C5	112.4 (4)	C30—C29—C28	117.7 (4)
C7—C6—C5	127.1 (4)	C30—C29—H29	121.2
C6—C7—C8	118.9 (4)	C28—C29—H29	121.2
C6—C7—H7	120.5	N6—C30—C29	123.5 (4)
C8—C7—H7	120.5	N6—C30—H30	118.2
C9—C8—C7	120.0 (4)	C29—C30—H30	118.2
C9—C8—H8	120.0	N7—C31—C32	178.0 (7)
C7—C8—H8	120.0	C34—C32—C33	117.9 (6)
C8—C9—C10	119.0 (4)	C34—C32—C31	121.7 (6)
C8—C9—H9	120.5	C33—C32—C31	120.3 (6)
C10—C9—H9	120.5	N8—C33—C32	178.5 (7)
N2—C10—C9	120.7 (4)	N9—C34—C32	179.3 (8)
N2—C10—C11	112.7 (4)	N10—C35—C36	178.2 (6)
C9—C10—C11	126.6 (4)	C37—C36—C35	119.5 (4)
N3—C11—C12	121.4 (4)	C37—C36—C38	119.6 (5)
N3—C11—C10	113.6 (4)	C35—C36—C38	120.8 (4)
C12—C11—C10	125.0 (4)	N11—C37—C36	179.2 (8)
C13—C12—C11	119.5 (4)	N12—C38—C36	179.2 (6)
C13—C12—H12	120.2		

### Hydrogen-bond geometry (Å, °)

**Table:**

*D*—H···*A*	*D*—H	H···*A*	*D*···*A*	*D*—H···*A*
C4—H4···N7	0.93	2.74	3.589 (7)	153
C8—H8···N9	0.93	2.71	3.347 (8)	126
C15—H15···N11	0.93	2.55	3.254 (6)	133
C1—H1···N12^i^	0.93	2.85	3.598 (6)	138
C23—H23···N11^i^	0.93	2.89	3.622 (6)	136
C2—H2···N10^ii^	0.93	2.77	3.670 (7)	164
C29—H29···N9^iii^	0.93	2.85	3.560 (8)	134
C17—H17···N8^iv^	0.93	2.65	3.395 (7)	137
C13—H13···N8^v^	0.93	2.65	3.379 (7)	136
C18—H18···N12^vi^	0.93	2.69	3.403 (6)	134
C22—H22···N10^vi^	0.93	2.51	3.231 (6)	134
C19—H19···N12^vii^	0.93	2.96	3.679 (6)	135
C22—H22···N12^vii^	0.93	2.92	3.645 (6)	136
C24—H24···N10^viii^	0.93	2.67	3.548 (6)	158
C27—H27···N10^viii^	0.93	2.57	3.350 (6)	142

Symmetry codes: (i) −*x*+1, −*y*+1, −*z*+2; (ii) *x*+1, *y*+1, *z*; (iii) −*x*+3/2, *y*−1/2, −*z*+3/2; (iv) −*x*+1/2, *y*−1/2, −*z*+3/2; (v) −*x*+1/2, *y*−3/2, −*z*+3/2; (vi) −*x*, −*y*+1, −*z*+2; (vii) *x*, *y*+1, *z*; (viii) *x*+1, *y*, *z*.
